# Novel Methods for Pulse Wave Velocity Measurement

**DOI:** 10.1007/s40846-015-0086-8

**Published:** 2015-10-14

**Authors:** Tânia Pereira, Carlos Correia, João Cardoso

**Affiliations:** Physics Department, Instrumentation Center, University of Coimbra, Rua Larga, 3004-516 Coimbra, Portugal

**Keywords:** Pulse wave velocity, Arterial stiffness, Commercial devices, Non-invasive methods, Measurement methods

## Abstract

The great incidence of cardiovascular (CV) diseases in the world spurs the search for new solutions to enable an early detection of pathological processes and provides more precise diagnosis based in multi-parameters assessment. The pulse wave velocity (PWV) is considered one of the most important clinical parameters for evaluate the CV risk, vascular adaptation, and therapeutic efficacy. Several studies were dedicated to find the relationship between PWV measurement and pathological status in different diseases, and proved the relevance of this parameter. The commercial devices dedicate to PWV estimation make a regional assessment (measured between two vessels), however a local measurement is more precise evaluation of artery condition, taking into account the differences in the structure of arteries. Moreover, the current devices present some limitations due to the contact nature. Emerging trends in CV monitoring are moving away from more invasive technologies to non-invasive and non-contact solutions. The great challenge is to explore the new instrumental solutions that allow the PWV assessment with fewer approximations for an accurately evaluation and relatively inexpensive techniques in order to be used in the clinical routine.

## Introduction

Cardiovascular diseases (CVDs) are a group of disorders that includes several heart diseases of circulatory system [1]. The World Health Organization refers the CVDs as the worldwide leading cause of death, resulting in a number of annually deceased people higher than from any other cause [1]. The improvements of diagnostics, treatments, medications, and surgical techniques were responsible for a significant decrease in total CVD mortality over the past few decades [1, 2]. Nonetheless, it is estimated that 17 million people died from CVD in 2011, which represents approximately 30 % of the global deaths, and approximately 23 million by 2030, to remain the single leading cause of death [3].

The great incidence of CVDs in the world spurred the search for new solutions to enable an early detection of pathological processes, to monitor the vital signals continuously, and to provide a more precise diagnosis based in multi-parameters assessment [4]. An accurate assessment of the CV system changes and the identification of risk factors is of utmost importance to avoid hospitalization and to reduce CVD morbidity and mortality rates [5]. The early detection based in multi-parameters of pathological condition is the key to the patient survival. Current CV risk evaluation systems are based on clinical judgment and traditional vital signals measurement, including heart rate, respiratory rate, blood pressure (BP), temperature and pulse oximetry. However, these vital signals are not sufficient to predict and evaluate the CVDs risk [1]. The appropriate management of classical risk factors such as (age, gender, smoking habits, hypertension, body mass index) and biological analysis (cholesterol, glucose, triglycerides, potassium, sodium) together with new biomarkers [pulse wave velocity (PWV), augmentation index] may represent a better method for more accurate diagnosis [6, 7]. Biomarkers are characteristics that are measured and evaluated as indicators of normal stages or pathogenic processes and responses to therapeutic interventions [8]. Efforts to identified new biomarkers have largely focused on the use of new measurements [9]. PWV is an emerging biomarker useful for CV risk stratification of patients, assessment of BP [10], vascular stiffness and for therapeutic effects and efficacy in clinical studies [11–13].

The arterial stiffness is the first vessels modification, responsible for several pathological processes, which can lead to CVDs. For this reason, the arterial elastic properties are used for risk stratification purposes in several populations. Recently, the European Society of Cardiology guidelines for the management of arterial hypertension suggested the measurement of PWV, considered the gold standard method for assessing arterial stiffness, as a tool to evaluate the arterial system damage, vascular adaptation, and therapeutic efficacy [6].

Another important application of PWV measurement is the indirect estimation of BP. It has been demonstrated that PWV is inversely related with BP and have been reported to be suitable for indirect BP measurements [14]. Important part of research studies in PWV was focused in the correlation between PWV and BP and on the development of a cuff-less continuous BP monitoring device [15–17]. Several models were proposed for BP estimation based in the PWV measurement [18], and they were based in the arterial wall mechanics and wave propagation in the arteries [18–20]. However, major progress on the algorithms to determine the BP using the PWV measurement is still required, like the calibration of pulse transit time (PTT, in units of ms) to BP (in units of mmHg) [21].

Apart from invasive methods, the PWV can also be measured using non-invasive, reproducible and relatively inexpensive techniques [7]. Many different waves have been used to determine the PWV, such as pressure wave, distension wave or flow wave. Commercial devices dedicate to PWV measurements make a regional assessment, i.e., the PWV is measured between two vessels. However the advantages of a local measurement are evident and some solutions are being exploited that enable this measurement in a short vessel segment.

There is an ever-increasing need and demand for novel and more efficient diagnostic tools to early detect CVDs. The great challenge is to explore new sensors and configurations that allow the PWV assessment with fewer approximations, leading to an accurate evaluation, and to develop relatively inexpensive techniques in order to be considered an interesting clinical solution. This paper aims to review the most relevant studies on PWV and introduce the discussion of advantages, disadvantages and applications of biomedical instrumentation for local versus regional PWV measurements.

## Pulse Wave Velocity: Regional Versus Local

PWV is defined as the velocity at which the pressure waves, generated by the systolic contraction of the heart, propagate along the arterial tree. The evaluation of PWV provides complementary information about the elastic properties of arterial system. The higher PWV corresponds to lower vessel distensibility and compliance and, therefore, to higher arterial stiffness. As the mechanical properties of the arterial walls change along the arterial system, from the large arteries to the periphery, PWV is also affected by these changes. The pulse waves travels through the arteries and its velocity depends of the vessel. PWV increases with the distance from the heart, along with the elastic condition of the arterial wall, which is affected by a variety of factors in the pathological process [22].

The regional PWV assessment is usually done in two different arteries (commonly the carotid and femoral arteries). Typical sites for pulse assessment are those where the pulsation can be easily felt, such as in the radial artery, in the common carotid artery, in the brachial or femoral arteries. The regional PWV only provides the average PWV over a long segment composed of arteries with different mechanical characteristics [22], consequently, regional measurements of PWV may mask the initial variations in arterial properties and could not evaluate biomechanical properties in a small segment and give no information about the position of the arterial abnormalities [23]. In the early stage of arterial stiffness, fibrous spots with small diameter are scattered on the arterial wall and, in the final stage, the arterial wall becomes homogeneously hard. The local PWV measurement is made on a short segment of an artery and represents an early diagnosis tool able to identify the local stiffness of the arterial wall [24, 25].

On the other hand, the regional estimation is based an external measurement of distance between the site where the pulse waveform was acquired. This coarse approximation of the distance between test points constituting an important disadvantage in the PWV assessment. The curvatures within the vasculature limit this approach, the problem of the distance measurement could be more severe in the presence of tortuous vessels that is not considered in an external measurement [26].

Carotid–femoral PWV is accepted as the ‘gold-standard’ measurement of arterial stiffness and it has been used to predict the CV events, assess the hypertensive, diabetics, and evaluate patients with chronic renal failure and coronary heart disease [6, 27]. The local PWV measurement is clinically important in local analyses of arterial wall properties to providing diagnostic information of biomechanical properties for local artery wall [23]. The mechanical characteristics of arterial vessels vary along the arterial tree and are differently affected by aging and disease [28]. On clinical application, in the early stage of atherosclerosis disease, the elastic properties will be affected locally and thus the functional properties of the local large arteries can be analysed with local assessment of PWV [28]. Recently studies have been dedicated to the local PWV measurement expecting to find diagnostic biomechanical information in some pathophysiologic conditions [28, 29].

The current methods dedicated to the pulse velocity estimation are based electromechanical solutions and all of them establish direct contact with the patient’s tissues at the artery site, which may distort the waveform. For instance, the probe needs to be placed over the widest pulsation area, and requires support from solid structures such as the bone. Several patients also feel quite a discomfort during the measurements, especially when their pulsation is not easily assessed (i.e., obese patients) [30]. The new solutions that are being developed try to overcome practical and technical limitations inherent to the currently used methods.

### Regional Pulse Wave Velocity

Framingham study created a multivariate risk scores to help the risk level of CVD classification. The first Framingham risk score was calculated by using information of disease, age, gender, diabetes, smoking, BP, and blood lipid concentrations. Several studies followed the Framingham work in order to improve and develop new guidelines for CVD risk management based on many others parameters that could amend the assessing individual risk. The multiple parameter risk response score is a useful tool to categorize patients in order to select the appropriate therapeutics. In the following studies main focus was given to the regional PWV as a predictive factor of CVD [31].

Woolam et al. estimated the PWV, using a piezoelectric crystal microphone to record the pressure pulse in radial and carotid arteries, in healthy subjects as ranged between 6.75 and 9.04 m s^−1^ and for diabetic subjects varying from 8.35 to 12.76 m s^−1^. A significant increase in the PWV was found in the diabetic subjects [32].

Isnard et al. studied, in a small population, the differences of PWV in normal subjects (8.9 ± 0.3 m s^−1^) and hypertension patients (11.8 ± 0.5 m s^−1^) [33].

Asmar et al. studied the PWV in 418 subjects and concluded that age and systolic pressure are the major determinants for the velocity of pulse propagation [34]. The differences between the PWV in normotensive, untreated hypertensive and treated subjects were studied. This study concluded that the age as a most important factor contributing to increase PWV, but did not suggest an important role of antihypertensive drug [35].

Blacher et al. developed a study in a hypertensive population, that showed the correlation of PWV with atherosclerosis alterations and constitutes a predictor of CV risk in hypertensive patients [36].

The relationship of PWV and CVDs was investigated in a hypertensive patients, and was verified higher PWV in patients with clinical vascular disease [37].

PWV was studied in subjects with 70–100 years old, which indicate that the increase in aortic PWV is an independent marker of CV risk in subjects with more than 70 years of age [38].

Laurent et al. performed a 9.3 years follow up study with 1980 patients, with the aim of determining if PWV is an independent predictor of CV mortality in hypertensive patients. The authors related that an increasing in 5 m s^−1^ in PWV is equivalent to an aging of 10 years in terms of increasing the mortality risk [39].

A study with a cohort of 1045 hypertensive patients with a mean age of 51 years and with a mean follow up period of 5.7 years, shows that relative risk of coronary event or any CV event increased with the increasing of PWV and evidences that PWV is an independent predictor of primary coronary events in patients with hypertension [40].

Another study, with random sample of 1678 subjects aged from 40 to 70 years, show similar values for PWV independently of gender, 10.8 ± 3.2 m s^−1^ for females and 11.8 ± 3.6 m s^−1^ for males. The authors refer the need to develop more sensitive techniques to measure the stiffness of various compartments of the arterial tree, which can be readily applied in routine clinical practice for risk stratification [41].

In the reference values for arterial stiffness collaboration, an European cross-sectional study, performed in 11,902 subjects, where PWV was assessed regionally (carotid–femoral) using several devices, it was showed that the obtained PWV values were lower in the group classified as normotensive (without CV risk factors subjects), from which the authors had established normal values of PWV. The results also demonstrated that PWV increases with age and hypertension severity. The normotensive group had a PWV mean distribution between 6.6 ± 0.8 m s^−1^ for subjects under 30 years old, and 11.7 ± 2.9 m s^−1^ for the age category above 70 years old [42].

A multicenter, observational study included 2200 Portuguese subjects, underwent clinical assessment and annual PWV measurement, demonstrated that PWV was significantly higher in individuals with CV events (11.76 ± 2.13 m s^−1^) than in those without events (10.01 ± 2.01 m s^−1^), evidencing the clinical relevance of PWV as a CV risk marker [43].

Several studies have been focused on the determination of reference PWV values in groups with healthy subjects and patients with CVDs, hypertension, diabetes or heart disease. Although most of these studies have been performed for regional PWV, the local measurement of the PWV is pertinent because of the arteriosclerosis local nature.

Table 1 gives a global vision of the results obtained in the multiple studies.

**Table 1:** Studies of PWV carotid–femoral measurement and reference values

Feature population/disease	Number of subjects	Age (years ± SD)	PWV (m s^−1^)	Devices	References
Healthy	15	33 ± 3	8.9 ± 0.3	Pulse transducer probe (Siemens Elema AB) synchronism with ECG	Isnard et al. [33]
Hypertensive subjects	16	38 ± 3	11.8 ± 0.5		
Healthy	418	46 ± 12	0.07 × SP + 0.09 × age − 4.3	TY-360 pressure transducer (Fukuda Co.)	Asmar et al. [34]
Normotensive subjects	124	45 ± 13	8.5 ± 1.5	Doppler unit (SEGA-M842, Oregon, USA) synchronism with ECG	Asmar et al. [35]
Untreated hypertensive patients	224	48 ± 11	11.8 ± 2.7		
Treated hypertension patients	164	59 ± 11	10.1 ± 2.6		
Hypertensive without atherosclerosis	530	57 ± 13	12.4 ± 2.6	Complior^®^	Blacher et al. [36]
Hypertensive with atherosclerosis	180	67 ± 12	14.9 ± 4.0		
Hypertensive without vascular disease	196	57 ± 13	12.4 ± 2.7	Complior^®^	Bortolotto et al. [37]
Hypertensive with vascular disease	40	62 ± 13	14.3 ± 4.0		
Subjects >70 years	141	87.1 ± 6.6	14.15 ± 3.11	Complior^®^	Meaume et al. [38]
Hypertensive patients	1980	50 ± 13	11.5 ± 63.4	Pulse transducer probes (electronics for medicine)	Laurent et al. [39]
Hypertensive without CV events	948	50 ± 12	11.4 ± 3.1	Pulse transducer probes (electronics for medicine)	Boutouyrie et al. [40]
Hypertensive with CV events	97	56 ± 13	12.8 ± 3.2		
Female	800	53.5	10.8 ± 3.2	Two piezoelectric pressure transducers (Hellige GmbH)	Willum-Hansen et al. [41]
Male	878	54.3	11.8 ± 3.6		
Female	1127	37	7.4	Complior^®^, SphygmoCor^®^, PulsePen^®^	Vermeersch et al. [42]
Male	1080	39	8.2		
Smoker	444	43	8.0		
No smoker	2207	38	7.8		
General population (52 % hypertensive, 11 % diabetic, 17 % smokers)	2200	46.33 ± 13.77	10.05 ± 2.03	Complior^®^	Maldonado et al. [43]

### Local Pulse Wave Velocity

Peripheral arteries are significantly stiffer than central arteries and this fact skews the interpretation of the real values of PWV. Besides that, the PWV in large central elastic arteries, such as the aorta or carotid, increases with age, whereas brachial or femoral arteries PWV do not increase so markedly [44]. Therefore, the large heterogeneity of the structure of arterial walls at different sites constitutes an important limitation of PWV regional measurement [45]. Furthermore, this convenient approach to analyse PWV on an artery segment avoids coarse approximations of the distance between the test points, constituting an important advance in PWV assessment. In fact, the carotid–femoral PWV assessment implies the measurement of the distance between carotid and femoral sites over the body surface. The accuracy of this measurement is markedly influenced by either the distance determination protocols or the presence of abdominal obesity [27]. In fact, the expert consensus document in arterial stiffness states that the PWV increases from 4 to 5 m s^−1^ in the ascending aorta, 5 to 6 m s^−1^ in the abdominal aorta and 8 to 9 m s^−1^ in the iliac and femoral arteries as the result from different studies [27]. A local PWV measurement technique is hence preferred [27].

Murgo et al. in 1980, measured the PWV in 18 patients by the catheterization in the ascending aorta and obtaining an average over all patients of 6.68 m s^−1^ [25]. In 1985, a smaller set of nine patients yielded a measured of 4.4 ± 0.4 m s^−1^ for the aortic root PWV, also during the catheterization [46].

The relationship between carotid–femoral and aortic PWV was studied in 107 patients [47]. The velocities were measured simultaneously during cardiac catheterization and proved the higher values obtained in carotid–femoral velocity (10.65 ± 2.29 m s^−1^) comparing to aortic velocity (8.78 ± 2.24 m s^−1^) [47].

Using an ultrasound (US) records, in 21 adults, the local PWV was measured in the left common carotid artery and values in range of 4–9 m s^−1^ were obtained [48], and it was verified that the velocity is increasing with age. In 2008, an experimental method, based on US data with higher frame rate, for the local determination of PWV in the carotid artery obtained values for PWV of 3–4 m s^−1^, however this study was only performed with one healthy subject with 36 years [49].

Another technique, using magnetic resonance, allows direct imaging of the thoracic and abdominal aorta. The PWV can be determined by measuring the pulse wave at two points in the vasculature and by estimating the path length of the pulse wave. The mean PWV obtained with this method in patients with diabetes mellitus, was 5.65 ± 0.75 m s^−1^ [26]. Table 2 summarized the main characteristics of the studies dedicated for local PWV.

**Table 2:** Studies of local PWV measurement and reference values

Feature population/disease	Number of subjects	Age (years ± SD)	PWV (m s^−1^)	Devices	References
Cardiac patients	18	34 ± 2	6.68 ± 0.32 Ascending aorta	Catheter (Millar Mikro-Tip, Millar Instruments, Houston, Texas)	Murgo et al. [25]
Cardiac patients	9	42 ± 5	4.4 ± 0.4 Aortic velocity	Catheter (Millar PC-786(K))	Latham et al. [46]
Cardiac patients	107	60.49 ± 8.31	8.78 ± 2.24 Aortic velocity	Catheter (6 Fr right Judkins catheter)	Podolec et al. [47]
Healthy	21	23–74	4–9 Carotid velocity	Ultrasound (GE Vingmed Ultrasound, Horten, Norway)	Rabben et al. [48]
Healthy	16	55 ± 7	5.65 ± 0.75	MRI (Gyroscan ACS/NT15, Philips, Best, The Netherlands)	Van der Meer et al. [26]
Type 2 diabetes mellitus	14	55 ± 8	6.83 ± 1.60 Aortic velocity		

## Devices

One of the major objectives of a CVD detection programme should be to identify the persons who have asymptomatic arterial disease in order to slow the progression of atherosclerotic disease and in particular to reduce the risk of clinical manifestations. The revolution in technology has a clear influence in the decision making of CV patients diagnostics, and can be applied in the early disease detection in asymptomatic patients. A large number of devices, to measure arterial stiffness are currently commercially available. The non-invasive measurement of arterial stiffness is usually accomplished by a set of devices that measure PWV and that provide capability to perform pulse pressure waveform analysis. The most common technique to non-invasively assess PWV is based on the acquisition of pulse waves generated by the systolic ejection at two distinct locations, separated by a distance *d*, by determining the time delay, or PTT, due to the pulse wave propagation along the arterial tree. The PWV parameter is then simply calculated as the linear ratio between d and the PTT [50].

Many different pulse waveforms have been used to assess PWV, such as pressure wave, distension wave or flow wave. In previous studies, the comparison between pressure and distension waveforms had shown that, these waves can be used interchangeably for many analyses due to their similar wave contour. The results of a number of studies have concluded that the distension waves are slightly less peaked than the pressure ones [51].

Some of the described equipment is able to determine regional PWV and others which are able to determine the local PWV measurement and are summarized in Table 2.

### Currently Devices

In order, to non-invasively assess the CV system several techniques have been developed. Some devices are routinely used in diagnostic work-up programmes in the clinic environment but they have rarely been used in the population as image screening tools such as magnetic resonance imaging (MRI) or ultrasonic sensors. However, these are usually seen as a costly technique and difficult to operate. More recently, new techniques have become available to detect new and several hemodynamic parameters. Pressure sensors, such as the tonometer, is considered the gold standard method for measuring PWV, but some operating skill is required to use this method which may influence the measurement. As a result, the tonometry technique of PWV assessment is usually used in research and clinical settings by well trained operators, but not widespread use in clinical or in ambulatory environments (Table 3).

**Table 3:** Devices and methods used to determine arterial stiffness

Methods	Measurements	Devices
Non-invasive	Regional PWV	PulsePen^®^
		Complior^®^
		SphygmoCor^®^
		Ultrasound
	Local PWV	Magnetic resonance image
		Ultrasound
Invasive	Local PWV	Angiography

Adapted from [52]

#### PulsePen^®^

The PulsePen (DiaTecne, Milan, Italy) is composed of one tonometer and integrated an electrocardiogram (ECG) unit. The delay between pulse waves is determined by applanation tonometry obtains at the carotid and femoral synchronized with the ECG R peak and the foot of the wave determined [53].

#### Complior^®^

The Complior^®^ (Colson, France) uses two dedicated piezoelectric pressure mechanotransducers directly applied to the skin in a simultaneous measurement of pressure pulses. The Complior was validated through a range of epidemiological studies that demonstrate the predictive value of PWV in CVDs. One probe is positioned at the common carotid artery, the central detection site, whereas the other probe is placed at the femoral artery site. However it is possible to determine the PWV in several pathways like carotid–femoral, carotid–brachial or femoral–dorsalis pedis without the need of ECG recordings [54].

#### SphygmoCor^®^

SphygmoCor^®^ (AtCor Medical, Sydney, Australia) analyzes the pulse wave of the carotid and femoral arteries, estimating the delay with respect to the ECG wave and calculating the PWV. SphygmoCor offers the possibility of carotid–femoral PWV measurements in two steps. The first step is used to simultaneously record carotid pulse wave and ECG, while the second step is the recording of femoral pulse wave and ECG. ECG recording during measurements is necessary for synchronization of carotid and femoral pulse wave times [54].

In the previously described techniques, PulsePen, Complior and SphygmoCor, the wave pathway is measured on the body surface. Distance measurements are taken with a measuring tape from the sternal notch to the carotid and femoral arteries at the sensor location. In fact, this measured distance is an estimation of the true distance travelled by the front wave and largely depends on body habitus [54], thus introducing a significant systematic error in the PWV estimation.

#### Arteriograph^®^

Arteriograph^®^ (TensioMed, Budapest, Hungary) does not measure propagation time from carotid to femoral waveform recordings or the distance between carotid and femoral arterial recording sites. The main principle of PWV estimation behind the Arteriograph device is to record oscillometric pressure curves based on plethysmography and register pulsatile pressure changes in an artery on the upper arm [54]. Since fluctuations in pulsatile pressure in the artery beneath the inflated pressure cuff induce periodic pressure changes in the inflated cuff, the oscillometric method measures these periodic pressure changes (oscillations) as an indirect measure for the pulsatile pressure changes in the artery beneath [55]. The pressure fluctuations in the brachial artery detected by the cuff are analyzed and the difference in time between the beginning of the first wave and the beginning of the second (reflected wave) is related to the distance from the jugulum to the symphysis, resulting in the PWV. The software of the Arteriograph decomposes the early, late systolic and diastolic waves and also determines the onset and the peaks of the waves [55]. This device uses an arm cuff like a sphygmomanometer, systolic and diastolic BPs are used for calibrate the system.

#### Photoplethysmography

Photoplethysmography is another non-invasive method for the detection of pressure waves propagated around the human body. The advantages of this method are: movement artefacts reduction [infrared (IR) probes lightly held against the skin without perturbing the physiological system], IR can then explore deep arteries according to the wavelength and to the distance between the light source and the detector. This latter characteristic is very interesting for assessing PWV in overweight populations. However this technique can only be applied to peripheral sites of the body. Pressure wave of blood undergoes changes in arterial tree, so that the waves obtained by this method don’t give reliable information from one central system.

The measurements of PWV in overweight and obese subjects may be of major interest in assessing CV risk. However, obesity is a well known factor of technical operator bias when assessing PWV. The image techniques are a commercial solution to acquire signals when the fatty deposit prevents the acquisition by other methods.

#### Ultrasound

The US technique allows the determination of the PWV by estimating the time delay between the diameter waveforms recorded simultaneously at two close positions along the vessels. The PWV is determined by the ratio of the temporal and the longitudinal diameter gradients. Alternatively the US may analyze the carotid and femoral waves simultaneously or separately using ECG synchronization [52]. These techniques depend on a reliable identification of the foot of the diameter waveforms and a sufficiently high sampling frequency. Another method from US measurements estimates local PWV as the ratio between change in flow and change in cross-sectional area during a cardiac cycle [48].

#### Magnetic Resonance Imaging

PWV measured by MRI allows the accurate assessment of the blood flow velocity with an enough temporal and spatial resolution to study the propagation of the aortic systolic flow wave [56]. MRI is a non-invasive technique that allows direct imaging of the thoracic and abdominal aorta without the use of geometric assumptions. The accurate and direct measurement of the path length of pulse waves in the proximal and distal aorta, even in the presence of a tortuous vessel, is a major advantage over other techniques [26].

The MRI allows the assessment of PWV, or other aortic vascular parameters (aortic distensibility, aortic compliance, aortic elastic modulus, and aortic stiffness index) [26]. Aortic PWV was calculated as Δ*x*/Δ*t*, where Δ*x* is the aortic path length between the two imaging levels and Δ*t* is the time delay between the arrival of the foot of the flow pulse wave at these levels [26, 57]. To extract ascending and descending aorta flow curves, aortic lumen contours are segmented. The intersection of the tangent line to the upstroke and the baseline were considered as the arrival time of the pulse wave [56].

#### Invasive Measurement

The best method for measuring PWV in aortic vessel is the threaded catheterization from a peripheral artery, during an angiography procedure. However, the invasive nature has several disadvantages and this method cannot be justified except during a cardiac diagnostic or vascular procedure [58].

### Non-commercial Devices: Novel Solutions Prototypes

The clinical application of novel non-invasive instrumentation can overcome practical and technical limitations inherent to the currently used methods such as arterial applanation tonometry, US and plethysmography that require physical contact of the probe with the patient and compress the artery throughout an entire cardiac cycle. These measurement techniques are affected by the Bernoulli effect that distorts the shape of the pulse curve itself [59, 60].

In tonometry, the probe needs to be placed over the widest pulsation area, and requires support from solid structures like the bone. For this reason, it is difficult to make valid measurements with applanation tonometry over the carotid artery, since they are usually involved in soft tissues [61]. Due to the contact nature, the exact positioning of the sensors is crucial for the correct measurement of PWV, and in consequence the quality of the recordings is strongly dependent of the operator. Several efforts have been done in the last decade to develop novel instrumentation which provides non-contact measurements. The following prototypes describe several systems based optical techniques that allow the monitoring of the PWV and other vital signals without contact with patient, which represent promising techniques for PWV measurement without distortion.

Meigas et al. presented an optical device based on self-mixing interferometry coupled with fiber optics to measure the carotid artery displacement. Along the optical sensor, an ECG was performed in order to determine pulse time delay [62]. Jukka et al. developed a self-mixing interferometry prototype applied to CV pulse measurements. The technique is used for pulse pressure waveform detection over the radial artery [63]. Scalise et al. proposed an optical procedure, with two optical head based Doppler vibrometer for measurements at long distances, suitable for monitoring the cardiac activity [64]. Mirko et al. developed a system based optical vibrocardiography for measure the regional PWV at the carotid and femoral site, with ECG signal for the synchronism [65]. Pereira et al. tested probes for local PWV measurement based coherent and non-coherent light, with two photodetectors placed 20 mm apart [66, 67].

The measurements of PWV in overweight and obese subjects may be of major interest in assessing CV risk. However, obesity is a well known factor which introduces difficulties in the assessment of arterial pulse wave for contact nature systems, the fatty deposits turns the signals acquisition a hard task to accomplish. The optical solutions, using light wavelengths that allows tissue penetration, captures the pulse waveform deep of the skin [68]. Yanlu Li et al. suggested a system with two laser Doppler vibrometry as a potential technique to measure the local carotid PWV in two locations along the common carotid artery from skin vibrations [69]. Recently, Adriaan Campo et al. presented an instrument with a double laser Doppler vibrometer to assess the local PWV at the carotid site [70].

### Comparison of Currently Methods

The current methods used to measurement the PWV can be divided in three main groups: imaging, non-imaging and optical methods. Each method has advantages and disadvantages (summarized in Table 4), and for this reason the PWV estimation remains a challenge for the engineers and clinicians.

**Table 4:** Advantages and disadvantages of methods to PWV measurement

Methods	Advantages	Disadvantages
Imaging	Direct measurement of the path length	Expensive technology
Non-imaging	More affordable (less expensive) and validated technology	Error associated with distance estimation, distortion in the acquisition signal, problems to access the signal in obese people, only allows a regional assessment
Optical	Low cost technology, measurement without contact, capability to acquire the signal in obese persons	Early stage of validation

The imaging methods, such US and MRI, take advantage of the pulse waves path length direct measurement and avoid the error induced by the coarse estimation of distance between the pulse pressure waveforms. However, these methods are very expensive and are not used in clinical routines. Non-imaging methods based in the pressure mechanotransducers are more affordable and extensively studied and validated in different populations, although, this method have problems on obese people signal acquisition, and have errors associated with distance estimation between the waveforms and originated with pressure exerted at the measurement site. The optical methods emerged to overcome the limitations of the previous methods. The optical solutions are low-cost, allows a non-contact measurement and the IR light allows the signal detection in obese people due to its penetration capability. These novel solutions are at an early stage of validation and nonetheless show very encouraging results.

## Conclusions

Although the arterial stiffness is accepted as one of the most useful parameter for assessment of the CV system, the discussion about its precise indirect measurement is open. The regional PWV was extensively studied in different groups of patients with distinct pathologies in order to establish the correlation between the PWV measurement with disease condition and the characteristics of population. However, considering the heterogeneous process of arterial stiffness, a diagnosis tool for measure the local stiffness of the arterial wall represents an important advance for an early and precise assessment of arterial condition.

Methods of PWV measurements have been refined since the early attempts at analysis, and have several relatively novel non-invasive methods for measuring the arterial pulse wave. Great importance has been done to novel solutions based non-contact techniques that allow overcoming the main limitations of current commercial devices. The optical sensors are an attractive instrumental solution in this kind of time assessment applications due to their truly non-contact nature that allows the measurement of local PWV in the carotid artery. The use of IR light, wavelengths which penetrates in the tissues, allows the arterial pulse wave acquisition in obesity cases. Moreover, its simple architecture, its time resolution and the lower production costs makes the use of optical prototypes very interesting and competitive. Good results were obtained in the preliminary tests of the developed optical prototypes. However, it is necessary further clinical researches with pathological populations in order to study the correlations with the local PWV. Future studies should provide the robust evidence of local PWV importance as a tool to determine the arterial stiffness in early stages.

An ideal method for arterial stiffness assessment would be able to measure the parameter directly, without any intermediate model or transfer function, to be an independent predictor for CV events, providing accurate information about the health condition of the patient, to be easy to use in daily clinical practice; allow a non-invasive and non-contact measurement and, to be validated in numerous studies with different groups of patients providing reference values that are accepted by the clinical community. The optical methods represent the non-invasive closest solution to this objective, allowing the measurement of PWV without contact, preserving the signal without interfering, and assessing the local PWV which is the earlier biomarker for arterial stiffness evaluation. The combination of this method with machine learning techniques would create an operator free system, thereby decreasing the error factor in signal detection and simplifying the acquisition.

With the development of new techniques that is become simpler, less expensive and more efficient, it is expected that the measurement of arterial stiffness will become an important part of clinical routine.
